# A Multistate Continuous Time-Inhomogeneous Markov Model for Assessing the CD4 Count Dynamics of HIV/AIDS Patients Undergoing Antiretroviral Therapy in KwaZulu-Natal, South Africa

**DOI:** 10.3390/ijerph22060848

**Published:** 2025-05-29

**Authors:** Chiedza Elvina Mashiri, Jesca Mercy Batidzirai, Retius Chifurira, Knowledge Chinhamu

**Affiliations:** 1 Department of Applied Mathematics and Statistics, Midlands State University, Gweru P Bag 9055, Zimbabwe; 2 School of Mathematics, Statistics and Computer Science, University of KwaZulu-Natal, Durban 4041, South Africa; chifurira@ukzn.ac.za (R.C.); chinhamu@ukzn.ac.za (K.C.); 3 School of Mathematics, Statistics and Computer Science, University of KwaZulu-Natal, Pietermaritzburg 3201, South Africa; batidzirai@ukzn.ac.za

**Keywords:** CD4 count, transitions, progression, multistate models

## Abstract

Monitoring CD4 count levels is essential for tracking the progression of HIV in patients. This study aimed to identify the key factors influencing HIV progression by incorporating time-varying factors and transition probabilities. The data for this study were obtained from the Centre for the AIDS Programme of Research in South Africa (CAPRISA), which enrolled 3325 patients aged 14 to 76 who initiated antiretroviral therapy (ART) and were followed up with between June 2004 and August 2013. The dataset included clinical, demographic, and treatment information to capture a comprehensive picture of HIV progression. To analyze the factors associated with HIV progression, this study employed time-inhomogeneous Markov models, which allow for incorporating covariates that change over time and transition probabilities. These models provided a robust framework to assess how various factors, such as CD4 count, viral load, and treatment adherence, evolve and influence disease progression. The results indicated that males had a significantly higher risk of moving from a normal (more than 500 cells/mm^3^) to mild state (351–500 cells/mm^3^) than females [HR: 1.614, 95% CI (1.281, 2.034)]. Rural patients had a significantly higher risk compared to urban patients of transiting from a mild state (351–500 cells/mm^3^) to an advanced state (200–350 cells/mm^3^) with a 95% confidence interval of (0.641, 1.009) [HR: 0.805, 95% CI (0.641, 1.009)]. The multistate model identified regimen, location, gender, and age as significant clinical variables influencing HIV progression. Rural patients and males showed slower transitions to CD4 count recovery. These findings provide valuable insights for disease management, treatment planning, and understanding the long-term prognosis for individuals living with HIV. Improving healthcare access, increasing educational efforts targeting men, reducing stigma, and fostering supportive environments can play a crucial role in enhancing CD4 count recovery and overall health outcomes for people living with HIV.

## 1. Introduction

The estimated overall HIV prevalence rate in the South African population is approximately 13.9% [1]. In 2022, the total number of people living with HIV (PLWHIV) in South Africa was estimated at approximately 8.45 million [2]. Among adults aged 15–49 years, the HIV prevalence rate was estimated at 19.6% of the population [2]. KwaZulu-Natal, the province with the highest HIV prevalence, had a rate of 27%. The CD4 count and viral load are considered the most important biomarkers for assessing disease stage and progression in patients with HIV [3,4].

A key characteristic of HIV is profound immunodeficiency, primarily resulting from a progressive decline in the quantity of CD4+ T cells [5]. The HIV-induced destruction of CD4+ T cells, whether directly or indirectly, leads to the loss of both HIV-specific and non-specific immune responses during the AIDS stage [6]. The CD4 lymphocyte count in individuals with HIV serves as a key indicator of disease progression and the risk of death from AIDS [7,8,9]. A lower CD4 count suggests significant immune system compromise, reducing the body’s ability to combat opportunistic infections [7,10].

Disease progression can be measured using time-homogeneous or time-inhomogeneous multistate Markov models. Transition probabilities are the basis of their differences. The transition probabilities of time-inhomogeneous models vary with time, whereas those of time-homogeneous models, such as Markov chains, are constant throughout time. The significance of time-inhomogeneous models lies in their ability to capture conditions that change over time.

Using viral loads and CD4 cell counts, time-homogeneous or time-inhomogeneous multistate Markov models assess immune recovery and disease progression. HIV disease progression and the factors influencing disease progression can be studied using longitudinal processes and joint multistate models. This study found that clinical characteristics, younger age, marital status, and educational attainment were significant factors that contributed to the progression of the disease [11]. Bidirectional transition rates were estimated using a semi-parametric time-homogenous multistate Markov model, and the covariate effects of age, gender, ART initiation period, and health facility level on the transition rates were evaluated [12].

To the best of our knowledge, few studies have applied time-inhomogeneous Markov models for CD4 count for South African datasets. Our study utilized a continuous time-inhomogeneous Markov chain model to determine significant factors in HIV progression, incorporating time-varying dynamics and transition probabilities that are not static. Reverse transitions were modelled to capture the arbitrary movements of patients among states. The results acquired here are compared with those in [13].

## 2. Materials and Methods

In this section, this study’s methodological framework was discussed, with a primary focus on the target population, sampling strategy, research design, statistical analysis, and explanation of key variables and elements.

### 2.1. Study Design

A prospective cohort study was conducted among patients with HIV initiating ART at the South African Centre for the AIDS Program of Research (CAPRISA) in KwaZulu-Natal, South Africa.

### 2.2. Study Population

This study’s two locations, Thekwini and Vulindlela, are urban and rural, respectively. Patients who missed three or more visits in a row and failed to be physically tracked were recorded as lost to follow-up and excluded from this study. This study included patients with CD4 count longitudinal data.

### 2.3. Data Collection and Preparation

The program enrolled 3325 patients for antiretroviral therapy initiation and follow-up between June 2004 and August 2013. CD4 counts, viral load, demographic characteristics, and medication regimens were recorded at baseline. The key variable of interest was the patients’ CD4 count (cells/mm^3^) which was followed up and registered on each visit. The CD4 count was categorized into four different states, namely normal (more than 500 cells/mm^3^), mild (351–500 cells/mm^3^), advanced (200–350 cells/mm^3^), and severe (fewer than 200 cells/mm^3^). CD4 count and viral load were taken at the beginning of this study and every six months or as needed for clinical reasons.

### 2.4. Statistical Analysis

**Figure 1:**
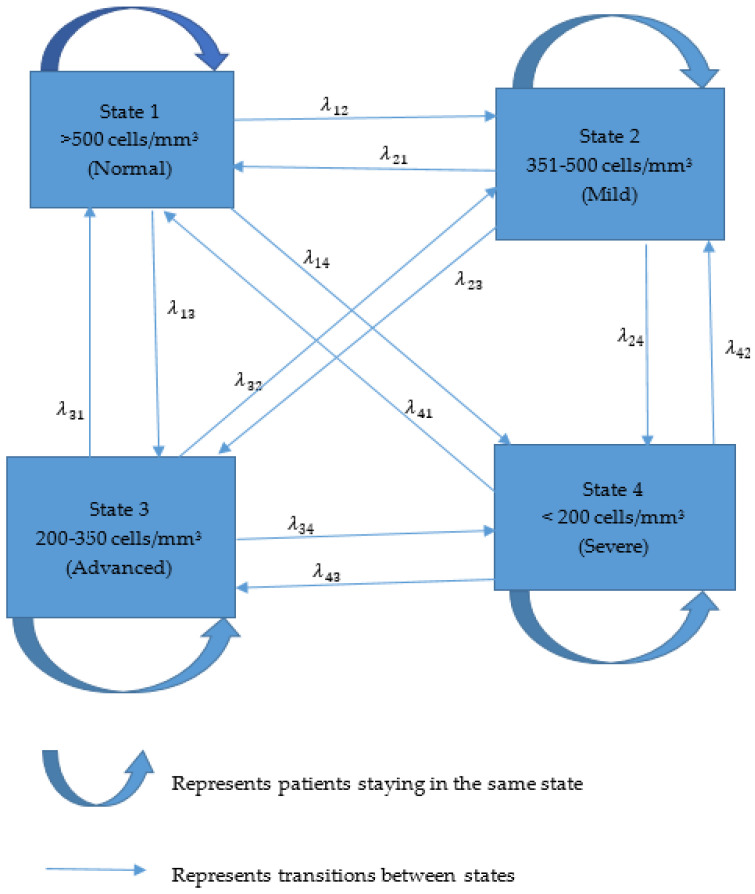
Flow diagram of states of patients who initiated ART.

A continuous time-inhomogeneous multistate Markov model was applied to analyze HIV/AIDS disease progression. The transitional time was calculated between each subsequent visit by a patient, and accumulated years were used in the Markov model. Reverse transitions were incorporated in the model, since patients moved arbitrarily to any state and no absorbing state was considered. The table on states shows the number of transitions between follow-up visits. The Markov model without a covariate was used to study the overall HIV/AIDS progression. Transition probabilities for one, five, and ten years described changes and the likelihood of moving from one state to another. The likelihood ratio test found significant variables that were used in the model. We initially started with a model with no covariates, then added variables individually and finally added all the variables. The Cox proportional hazard approach was utilized in the multistate model. Multistate models whose Hessian matrix was not positive definite were excluded from this study. The categorical variables employed in the model were age (≤44 years, >44 years), regimen (first-line treatment and second-line treatment), site (urban and rural), and gender (males and females). According to the WHO (2015), patients that are 44 years old or younger are referred to as young. An analysis was conducted using the msm package from R-studio. The transient states in the multistate Markov model are shown in Figure 1.

### 2.5. Statistical Model

#### 2.5.1. Multistate Markov Model

A multistate process is a stochastic process (X(t), t∈T) with a finite transient state space S={1,2,3,4}. T=[0, τ], τ<∞ is the time interval and the value of the process at the time t that the state occupied at that time. Suppose X(t)=r is the state of a patient at any time t; then, the intensity with which the patient moves to the state s during the instantaneous interval (t, t+∆t) is given by

(1) $$\lambda_{rs}\left(t\right)=\underset{∆t\to 0}{\lim}\frac{P\left(X\left(t+∆t\right)=s|X\left(t\right)=r\right)}{∆t}\;r,s=1,\;\;2,\;3,\;4$$

Equation (1) constitutes the $rs^{th}$ element of the transition intensity matrix $\Lambda ={\left[\lambda_{rs}\right]}_{4x4}$. The intensity matrix Λ is defined such that each element $\lambda_{rs}$ represents the rate at which a patient moves from state r to state s during an instantaneous interval ∆t.

#### 2.5.2. Properties of Transition Intensity Matrix

1. Non-negativity: For all r and s, $\lambda_{rs}\geq 0$ for all r≠s.

This shows that the transition intensities from one state to another cannot be negative.

2. Row Sums: The sum of each row must equal zero: $\underset{s\epsilon S}{\sum }\lambda_{rs}=0$ for each state r.

This ensures that the total rate of leaving state r is equal to the sum of rates to all other states.

The transition probabilities quantify the likelihood that an individual moves from one state to another over a specified time interval. The transition probability matrix P(t) can be computed from the transition intensity matrix Q using the Chapman–Kolmogorov equations.

#### 2.5.3. Assumptions of Multistate Markov Model in Statistical Formulation

1. The future state depends only on the present state, not on the past.
2. Transitions between states can occur at any time, not just at discrete intervals.
3. The probability of transitioning from one state to another change over time.
4. The model considers a finite number of possible states.
5. The probability of a transition depends only on the current state and not on the history of states.

#### 2.5.4. Chapman–Kolmogorov Equations

The Chapman–Kolmogorov differential equations describe how to compute the transition probabilities over time. If P(t) represents the likelihood of being in state r at time t given that the individual was in state s at time 0, then

(2) $$P\left(t\right)=e^{Qt}$$

where the variables are defined as follows:

$e^{Qt}$ is the matrix exponential of Qt. Q is a matrix of intensities whose rows sum to zero. The estimated transition intensity matrix is used to fit a multistate model to the data.

The estimates of transition intensities are used to compute the transition probability matrix $P\left(t\right)={\left[P_{rs}\left(t\right)\right]}_{4x4}$, by solving the Chapman–Kolmogorov differential equations, where $P_{rs}\left(t\right)$ is the probability of a patient with HIV/AIDS being in a state s at a time (t+∆t) given that the subject was at a state r at time t, which is mathematically represented as

(3) $$P_{rs}\left(t\right)=P\left(X\left(t+∆t\right)=s|X\left(t\right)=r\right)$$

The transition parameters are estimated using various statistical techniques, such as the Maximum Likelihood Estimation (MLE) and Bayesian methods. This study utilized the Maximum Likelihood Estimation method to estimate the transition intensities, allowing covariates to influence the intensities. Individual-specific or time-dependent covariates can be included in the model to explain variations in transition intensities.

A time-inhomogeneous Markov model is a Markov model where the transition probabilities between states can change over time. This model evaluates the likelihood of moving from one state to another depending on the specific time point or interval. This model consists of a finite or infinite set of states. The transition probabilities from state i to state j can vary with time t. This model is represented as follows:

(4) $$P_{ij}\left(t\right)=P\left(X_{n+1}=j|X_{n}=i,\;T_{n}=t\right)$$

where $X_{n}$ is the state at a time t.

#### 2.5.5. Mean Sojourn Time

The mean sojourn time represents the expected duration or average time that a Markov chain spends in a particular state before transitioning to a different state. For a time-inhomogeneous Markov chain, the mean sojourn time in state i at time t is denoted as $M_{i}\left(t\right)$. The cumulative distribution function (CDF) is used to calculate the sojourn time in state i at time t and is written as follows:

(5) $$F_{i}\left(t,s\right)=P(X\left(t+s\right)\neq i|X\left(t\right)=i)$$

Equation (5) represents the probability that the Markov chain leaves state i within s units of time, given that it was in state i at time t. The mean sojourn time in state i at time t is then given by the following:

(6) $$M_{i}\left(t\right)=\int_{0}^{\infty }\left(1-F_{i}\left(t,s\right)\right)ds$$

The integral represents the expected time that the Markov chain remains in state i at time t before transitioning to a different state. A higher mean sojourn time indicates that the Markov chain tends to stay longer in that state before transitioning. The mean sojourn time characterizes the dynamics and behaviour of the Markov chain, as it provides insights into the typical durations of visits to different states.

#### 2.5.6. Modelling the Intensity Function Using the Cox Proportional Hazard Function

The Cox proportional hazard model expresses the hazard function h(t) for an individual as follows:

(7) $$\lambda_{rs}\left(t\right)=\lambda_{rs0}\left(t\right)e^{\beta_{rs1}X_{rs1}+\cdots ..+\beta_{rsk}X_{rsk}}$$

where

$\lambda_{rs0}\left(t\right)$ is the baseline hazard function associated with the transition of type rs (the hazard for an individual with all covariates equal to zero).

$e^{\beta_{rs1}X_{rs1}}$ is the exponential function that represents the effects of covariates $X_{rs1}$ on the hazard, and $\beta_{rs1}$ represents the coefficients to be estimated. The covariates are assumed to be constant over time. The Cox proportional hazard model permits the integration of covariates that may impact the risk of moving between states, offering more detailed insights into the survival process. The msm R package (R version 4.1.2) uses the Maximum Likelihood Estimation method for the multistate model’s parameter estimation and transition probabilities.

### 2.6. Model Diagnostics

The likelihood ratio test is a statistical method used for model validation. The calculation for this test is defined as $-2\left(log\left(l_{0}\right)-log\left(l_{1}\right)\right)$, where $l_{0}$ is a model with no covariates, and $l_{1}$ is a full model with covariates, which follows a chi-squared distribution with k degrees of freedom with a 5% level of significance. The aim of this test is to obtain a model of simple parsimony.

## 3. Results

Table 1 shows the descriptive statistics calculated for 3325 study subjects which were followed up with, of which 2148 (65%) were female, and 1770 (53%) were from rural areas. There were 2515 (76%) patients who had no signs of tuberculosis, and 810 (24%) had tuberculosis before initiating ART. The number of patients that were older than 44 was 445 (13%), and the number of those that were 44 or younger was 2880 (87%).

Table 2 displays the number of transitions between follow-up visits. The following transitions occurred: mild to severe (113), advanced to normal (363), severe to normal (98), and normal to severe (48).

The Markov model without a covariate was used to study the overall HIV/AIDS progression, and the results are presented in Table 3. Patients in a normal state (more than 500 cells/mm^3^) are 16.5 times (0.595/0.036) more likely to move from mild to severe states. Transitioning from a mild state to a normal state resulted in a 1.6 times (1.215/0.781) greater chance to transition to an advanced state. Patients in an advanced state were 2.1 times more likely to move from a mild state to a normal state, that is, the recovery state. Once a patient is in the severe state, they are 262 times (1.572/0.006) more likely to move to an advanced state than to a normal state. A patient who is in a severe state is three times (0.006/0.002) more likely to move to a normal state than a mild state. The negative estimates suggest that a patient has a smaller chance of staying in the same state.

The transition probabilities for one, five, and ten years are presented in Table 4. At the end of a year, patients in a normal condition have a 63% probability of remaining in that state. The chances of progressing to mild, advanced, and severe (fewer than 200 cells/mm^3^) were 22%, 11%, and 4%, respectively. Within the first year of starting ART, patients with mild conditions had a 41% chance of returning to a normal state, a 30% chance of remaining in the same state, and an 8% chance of moving to a severe state. Patients in a severe state had a 31% likelihood of remaining in their current condition, a 40% risk of progressing to an advanced level, and a 9% chance of returning to a normal state (more than 500 cells/mm^3^). Over five years, patients in the normal state had a 43% chance of remaining the same, a 24% likelihood of moving to a mild state, and a 10% chance of progressing to a severe state. Those in a mild state had a 23% probability of remaining the same while transitioning to an advanced state, with a 42% chance of reversing to normal and an 11% chance of transitioning to a severe state. Patients in both advanced and severe states have probabilities of 41% and 40%, respectively, of reverting to a normal state after five years. Additionally, individuals in a normal state have a 42% likelihood of staying in that state, while those in mild, advanced, and severe states also have a 42% chance of transitioning to a normal state following a period of ten years.

The estimates of the mean sojourn time along with 95% confidence intervals are shown in Table 5. The average time patients spent in a normal state (more than 500 cells/mm^3^) is 1.41 years with a 95% confidence interval of (1.30, 1.54). The average period spent in a mild state (351–500 cells/mm^3^) was 0.48 years (95% CI: 0.45, 0.50) before moving to another state. The average sojourn time patients spent in a severe state (fewer than 200 cells/mm^3^) was 0.63 years with a confidence interval of (0.60, 0.66) before transitioning to other states.

Table 6 shows that the model comparison presents various combinations of covariates and their corresponding likelihood ratios and *p*-values. The investigation of disease progression was broadened to incorporate covariates through multistate modelling employing the Cox proportional hazard method. We commenced with a base model that contained no covariates. Each variable was progressively added to the initial model, starting with one, then two, then three, and eventually all four at once. The combinations of variables that resulted in a Hessian matrix that was not positive definite were omitted from the final model. Likelihood ratio tests were conducted for each model at various levels. The final multivariable model consisted of significant variables: regimen, site, age, and gender.

The hazard ratios (HRs) from the Cox proportional hazard function for each covariate (regimen, site, age, and gender) on each transition along with the 95% confidence intervals for transitions in a one-year interval are presented in Table 7. The final model comprised combined variables with a positive definite Hessian matrix. The transition from a normal to a mild state was significantly associated with age, site, gender, and regimen. Males were 1.6 times more likely to move from a normal to mild state compared to females [HR 1.614; 95% CI (1.281, 2.034)] within a year. Rural patients had a hazard ratio value of 81% compared to urban patients transiting from a mild state to an advanced state with a 95% confidence interval of (0.641, 1.009). Patients that were older than 44 had higher chances of transitioning from an advanced to severe state compared to the younger group [HR 0.926; 95% CI (0.798, 1.075]. The significant transition from a severe to advanced state was associated with covariates regimen, site, age, and gender. Their hazard ratios and 95% confidence intervals were [HR 0.743; 95% CI (0.655, 0.844)], [HR 0.730; 95% CI (0.657, 0.811)], [HR 0.915; 95% CI (0.841, 0.995)], and [HR 0.863; 95% CI (0.779, 0.956)], respectively.

## 4. Discussion

We studied the HIV/AIDS progression of patients in normal, mild, severe, and advanced states. Patients with a CD4 count of fewer than 200 cells/mm^3^ had lower chances of CD4 count recovery (to more than 500 cells/mm^3^) than those in other states, leading to a risk of immune deterioration. Previous authors supported the same results [14,15,16,17,18]. The likelihood of moving from the normal to severe state (CD4 count of fewer than 200 cells/mm^3^) was minimal. This was evidenced by low transition probabilities, indicating that it is less likely for patients in the normal state to move to the severe state over a given time interval. The risk of developing severe health conditions is low for patients not experiencing severe HIV symptoms. Patients with a CD4 count in the normal state had relatively low chances of their condition deteriorating to a severe state [15,19]. The natural progression of HIV disease may involve gradual declines in CD4 count over time. The transition from the normal to severe state may be less likely to occur with early intervention and treatment adherence. Low transition probabilities were observed in five- and ten-year periods, which showed lower chances of moving from normal to severe. The results reflected a stable health status and long-term health prospects for the patients over ten years. Patients who had transitioned from a severe state to a normal state had an increased transition probability during the five- and ten-year periods compared to those in their first year of ART initiation. Patients who adhered to ART and were monitored for a long period had increased chances of CD4 count recovery from severe health states. The transition from an advanced state (200–350 cell/mm^3^) to the recovery state was slightly low in the first year of ART initiation and increased in the five- and ten-year periods. Patients’ stability was evidenced by increased transition probabilities in the mild state (351–500 cells/mm^3^) to the normal state from the first year and the five- and ten-year periods of ART initiation [20,21].

In terms of the covariate effect, HIV/AIDS patients that were older than 44 had higher chances of experiencing backwards and forward progression, through some of them had significant hazard ratios. We noted a significant transition from a mild state to a normal state for patients older than 44. Older individuals may develop a more robust adaptive immune system over time, which can help maintain a relatively stable CD4 count and delay the progression to the severe state. The adaptive immune response can be further enhanced by consistent HIV treatment and effective viral suppression [22,23,24,25]. Older HIV patients may have better adherence to ART regimens, as they tend to have a greater understanding of the importance of consistent medication intake compared to the young generation. Healthcare providers should closely monitor older HIV patients, thereby identifying and addressing potential risks that could accelerate disease progression. According to [26,27], older patients have a reduced risk of non-adherence to ART than their younger counterparts.

Our study showed that female HIV patients generally have higher transition probabilities from the mild state to the recovery state compared to their male counterparts. Female patients living with HIV are more likely to experience an improvement in their CD4 count, reaching the normal state more often compared to males. Women may be more likely to engage in regular healthcare visits and adhere to antiretroviral therapy that can facilitate the transition to a normal state. Females are more likely to be diagnosed with HIV earlier in the disease course [28,29], leading to the earlier initiation of ART and a better chance of reaching the recovery state. Our results concurred with other findings that male patients transitioned from normal states to severe states more often compared to female patients [12,30,31]

The results showed that HIV patients living in rural areas have lower transition probabilities from the mild state to the normal state (CD4 count ≥ 500 cells/mm^3^) compared to those living in urban areas. Rural patients have a lower risk of experiencing CD4 count recovery and reaching the recovery state. This might be caused by limited access to specialized healthcare services, such as HIV clinics, antiretroviral therapy (ART) providers, and experienced healthcare professionals. Patients usually walk long distances to these essential services and may have transportation barriers, which may contribute to rural patients’ inadequate ability to receive timely and consistent care, which is crucial for achieving CD4 count recovery [32,33,34,35]. Poor adherence and loss to follow-up can significantly reduce the likelihood of CD4 count recovery for rural HIV patients. The limited availability of specialized HIV care services, such as viral load monitoring, CD4 count testing, and access to new or innovative ART regimens, can affect the CD4 count recovery of rural patients [36]. Stigma and a lack of education in rural areas can lead to late diagnoses, resulting in lower CD4 counts and more advanced stages of HIV [37,38,39].

Patients on the first-line ART regimen generally had higher transition probabilities of moving from the mild to the normal state compared to patients on the second-line ART regimen. Viral suppression and immune reconstitution were successfully achieved with first-line ART, which facilitated the transition to the normal state. First-line ART regimens are more effective in achieving and maintaining viral suppression, which is a crucial factor in preventing disease progression and promoting CD4 count recovery [40]. Consistent adherence to first-line ART can help patients reach and sustain undetectable viral loads, which is associated with better immunological outcomes [41].

One limitation of this study was that some patients only came once for ART initiation and never came for routine check-ups; therefore, such data cannot be used in multistate models. Loss to follow-up data disproportionately affect demographic groups and biasness on transition probability estimates. Multistate models require longitudinal data only for analysis.

## 5. Conclusions

The significant variables incorporated in the multistate model were regimen, site, gender, and age. Patients on the first line of treatment transitioned from a mild state to a normal state in terms of CD4 count compared to second-line patients. Patients given the first-line treatment had increased viral load suppression. Rural area patients had low levels of healthcare-seeking behaviours compared to urban patients. Older patients and females showed better treatment adherence. These results can be valuable for disease management, treatment planning, and understanding the long-term prognosis of individuals living with HIV. We recommend educational programs tailored specifically for males regarding ART adherence, which can significantly enhance their chances of improving CD4 counts and achieving better health outcomes.

## Figures and Tables

**Table 1:** Descriptive characteristics of study variables at ART initiation (N = 3325).

Variables	Category	Number (%)
Age	≤44	2880 (87%)
	>44	445 (13%)
Tuberculosis	Initiated ART with TB	810 (24%)
	Initiated ART without TB	2515 (76%)
Site	Eswatini (Urban)	1555 (47%)
	Vulindlela (Rural)	1770 (53%)
Gender	Female	2148 (65%)
	Male	1177 (35%)
Initial State	1	57 (1%)
	2	126 (4%)
	3	723 (22%)
	4	2419 (73%)

**Table 2:** Number of observed transitions between states (rows to columns).

To From	Normal	Mild	Advanced	Severe
Normal	2058	444	122	48
Mild	967	1252	527	113
Advanced	363	1444	2821	667
Severe	98	383	2045	2896

**Table 3:** Estimates of one-step transition intensities (95% confidence interval) for time-inhomogeneous Markov model.

States	Normal Est (95% CI)	Mild Est (95% CI)	Advanced Est (95% CI)	Severe Est (95% CI)
Normal	−0.708 (−0.772, −0.650)	0.595 (0.535, 0.661)	0.078 (0.055, 0.112)	0.036 (0.024, 0.053)
Mild	1.215 (1.143, 1.292)	−2.105 (−2.218, −1.997)	0.781 (0.704, 0.866)	0.109 (0.082, 0.145)
Advanced	0.003 (0, 0.073)	1.170 (1.113, 1.230)	−1.738 (−1.817, −1.663)	0.566 (0.518, 0.619)
Severe	0.006 (0.002, 0.019)	0.002 (0, 0.038)	1.572 (1.499, 1.648)	−1.580 (−1.655, −1.508)

**Table 4:** Estimated transition probabilities between states (rows to columns) of HIV/AIDS patients.

States	1 (Normal)	2 (Mild)	3 (Advanced)	4 (Severe)
1 year				
1 (Normal)	0.63110667	0.2152040	0.1110817	0.04260763
2 (Mild)	0.40843882	0.2978107	0.2128788	0.08087167
3 (Advanced)	0.19980597	0.2688711	0.3726544	0.15866850
4 (Severe)	0.09675155	0.1903095	0.3986161	0.31432282
5 years				
1 (Normal)	0.4302635	0.2443956	0.2211111	0.1042298
2 (Mild)	0.4228654	0.2253476	0.2253476	0.1069569
3 (Advanced)	0.4128209	0.2454068	0.2311008	0.1106715
4 (Severe)	0.4042744	0.2458729	0.2359979	0.1138548
10 years				
1 (Normal)	0.4218898	0.2448793	0.2259069	0.1073239
2 (Mild)	0.4217419 8	0.244887	0.2259917	0.1073786
3 (Advanced)	0.4215407	0.2448994	0.2261069	0.1074530
4 (Severe)	0.4213691	0.2449093	0.2262052	0.1075164

**Table 5:** Mean sojourn time.

State	Mean Sojourn Time	
	Estimate	SE (95% CI)
Normal	1.4118	0.0620 (1.2954, 1.5387)
Mild	0.4753	0.0127 (0.4510, 0.5010)
Advanced	0.5751	0.0129 (0.5504, 0.6010)
Severe	0.6330	0.0151 (0.6042, 0.6633)

**Table 6:** Likelihood ratio (LR) test for model selection.

Covariates	“−“2”∗(logLikelihood)	LR Test	DF	*p*-Value
No covariates	33,844			
Site	33,562	282	24	<0.0001
Tuberculosis (TB)	33,604	240	24	<0.0001
Gender	33,686	158	24	<0.0001
Age	33,772	72	24	<0.0001
Regimen	33,782	62	24	<0.0001
TB + Age	33,532	312	36	<0.0001
TB + Age + Gender	33,220	624	48	<0.0001
Regimen + Site + Age + Gender	33,246	598	60	<0.0001

Note: LR means Likelihood Ratio Test, DF refers to Degrees of Freedom, *p*-Value refers to the probability value.

**Table 7:** Estimates of hazard ratios and 95% confidence intervals for parameters of joint multistate model.

	Variables			
	Regimen (Ref: First-Line Treatment) Second-Line Treatment	Site (Ref: Urban) Rural	Age (Ref: ≤44) >44	Gender (Ref: Female) Male
Transitions	HR (95%CI)	HR (95%CI)	HR (95%CI)	HR (95%CI)
Normal–Mild	1.310 (1.031, 1.664)	1.079 (0.821, 1.420)	1.062 (0.906, 1.246)	1.614 (1.281, 2.034)
Normal–Advanced	0.022 (0, 238.67)	0.0009 (0, 5.786)	1.648 (0.856, 3.174)	3.125 (1.583, 6.185)
Normal–Severe	0.121 (0.013, 1.066)	0.071 (0.025, 0.200)	0.743 (0.358, 1.543)	0.916 (0.311, 2.696)
Mild–Normal	1.180 (0.997, 1.40)	1.142 (0.974, 1.338)	0.957 (0.865, 1.059)	0.909 (0.780, 1.060)
Mild–Advanced	1.174 (0.093, 1.482)	0.805 (0.641, 1.009)	1.089 (0.933, 1.059)	1.126 (0.922, 1.376)
Mild–Severe	0.423 (0.179, 0.996)	0.030 (0.002, 0.427)	0.750 (0.447, 1.259)	2.528 (1.354, 4.719)
Advanced–Normal	0.078 (0, 324.064)	0.011 (0, 20.422)	0.428 (0.074, 2.495)	0.301 (0, 292.98)
Advanced–Mild	0.942 (0.818, 1.084)	0.909 (0.808, 1.021)	0.842 (0.774, 0.916)	0.804 (0.718, 0.900)
Advanced–Severe	1.373 (1.117, 1.688)	0.596 (0.494, 0.719)	0.926 (0.798, 1.075)	1.358 (1.136, 1.623)
Severe–Normal	1.005 (0.094, 10.741)	0.093 (0, 98.023)	5.150 (0.080, 332.34)	1.322 (0.114, 15.384)
Severe–Mild	7 (0.038, 1280.472)	0.192 (0.006, 6.071)	0.009 (0, 2.591)	0.084 (0, 1062.727)
Severe–Advanced	0.743 (0.655, 0.844)	0.730 (0.657, 0.811)	0.915 (0.841, 0.995)	0.863 (0.779, 0.956)

## Data Availability

CAPRISA has an established procedure to make its research data more broadly available. Information on the process for requesting and obtaining data is available on the CAPRISA website (www.caprisa.org). The datasets used for the analyses for the CAPRISA research article that has been published can be obtained by request to any investigator through an online request lodged on the CAPRISA website for the CAPRISA Scientific Review Committee to review, and once approved, the datasets, study protocol, and statistical analysis plan will be made available to the interested investigators making the request.

## Abbreviations

HIV: Human Immunodeficiency Virus; ART: antiretroviral therapy; CAPRISA: Centre for the AIDS Programme of Research in South Africa; CI: confidence interval; HR: hazard ratio.
